# Open Latarjet Versus Arthroscopic Bankart Repair for the Treatment of Traumatic Anterior Shoulder Instability in High-Demand Patients With Minimal Glenoid Bone Loss

**DOI:** 10.7759/cureus.37127

**Published:** 2023-04-04

**Authors:** Ahmed Genena, Mohamed Hashem, Ahmed Waly, Mohamed O Hegazy

**Affiliations:** 1 Trauma and Orthopaedics, Faculty of Medicine, Helwan University, Cairo, EGY; 2 Trauma and Orthopaedics, James Paget University Hospital, Great Yarmouth, GBR; 3 Trauma and Orthopaedics, Faculty of Medicine, Alexandria University, Alexandria, EGY; 4 Trauma and Orthopaedics, Faculty of Medicine, Benha University, Cairo, EGY

**Keywords:** minimal glenoid bone loss, high demand patients, arthroscopic bankart, open latarjet, shoulder instability

## Abstract

Background: This study compared the clinical outcomes and return to sports/work between open Latarjet and arthroscopic Bankart repair in high-demand patients with traumatic anterior shoulder instability with minimal glenoid bone loss.

Methods: We prospectively recruited 30 patients and randomised them to either open Latarjet or arthroscopic Bankart. The mean duration of follow-up in our study was 13.27 months±2.70. All patients were males with a mean age at surgery of 28.6 years (range, 18-41 years).

Results: The overall mean for the Rowe score in the 30 patients increased from 33.5±14 points preoperatively to 79.6±18 points. However, there was no statistically significant difference in the postoperative ROM (range of motion) and Rowe score among the Bankart and Latarjet groups.

The main finding in our study was the time to return to sports/work which was significantly lower in the Latarjet group (5.2 months) compared to the Bankart group (seven months).

Conclusions: Open Latarjet is considered a more invasive and non-anatomical procedure, however, it is less costly with a shorter time to return to sports/work compared to the Bankart procedure, which is very crucial for high-demand patients, especially the competitive athletes targeting an early return to sports at the same pre-injury level with minimal incidence of recurrence, making the surgeon’s choice very challenging.

## Introduction

The shoulder is the most mobile joint in humans. The shoulder girdle is composed of the clavicle and the scapula, which articulates with the proximal humerus of the upper limb. Four joints are present in the shoulder: glenohumeral (GH), acromioclavicular (AC), sternoclavicular (SC) and scapulothoracic joints. This wide range of movement predisposes to high susceptibility to dislocation. Shoulder dislocation represents 50% of all joint dislocations, particularly in young people [1,2]. Anterior glenohumeral instability is a common problem in the athletic, young population, with a rate of 12 per 100,000 athlete exposure [3]. In addition, athletes taking part in contact sports are more prone to high-velocity collisions and positions making their shoulders susceptible to injury in comparison to non-contact athletes [4].

The surgical management of recurrent instability was first described by Bankart in 1938 [5]. Open Bankart stabilization was considered the gold standard treatment with earlier studies showing better results compared to arthroscopic stabilization. However, arthroscopic techniques have been evolving and arthroscopic repair with suture anchors now attains equivalent results to open repair in the treatment of Bankart lesions [6].

Later, with a better understanding of the changes in the patho-anatomy with recurrent shoulder instability, the focus has been shifted toward bony stabilization versus soft tissue procedures as humeral and/or glenoid bone lesions have been demonstrated to occur in around 90% of shoulders with recurrent instability. Failure to treat these bony defects can lead to a poor outcome. A bony glenoid defect of 21% has been shown to jeopardize shoulder stability [7].

Michel Latarjet [8] in 1954 represented his shoulder stabilization technique, where the horizontal limb of the osteotomized coracoid process is transposed to the anteroinferior rim of the glenoid to restore the glenoid’s anteroposterior diameter. In addition to the sling effect which involves the interaction between the lower fibers of the subscapularis and the conjoint tendon while the arm is in the position of ER (external rotation) and abduction.

Evidence-based data support the efficacy of both surgeries; arthroscopic Bankart and Latarjet, and the surgeons’ choice plays a major role in opting for one option over the other. Advocates of the Latarjet procedure defend their choice based on the lower rate of recurrence and shorter time to return to the patients’ preinjury sports levels. On the flip side, arthroscopic Bankart restores the normal shoulder anatomy as well as maintains the range of motion (ROM) [9].

The Latarjet procedure proved to be effective in dealing with anterior recurrent shoulder instability with severe glenoid bony loss, as a primary or revision procedure [10]. However, there is scant information in the literature in terms of optimal treatment of high-demand patients with minimal glenoid bone loss (<20%) [11-14].

## Materials and methods

This study received ethical approval (IRB approval number 11-2019) from the Research Ethics Committee (REC) for Human Subject Research at the Faculty of Medicine, Helwan University and informed consent was taken from all participants.

Patient selection

This prospective randomized trial was done at our institution between February 2019 and July 2021. Thirty patients were equally randomized into either arthroscopic Bankart or open Latarjet. All patients met the inclusion criteria (Table 1).

**Table 1:** Inclusion and exclusion criteria

Inclusion criteria	Exclusion criteria
Traumatic anterior shoulder instability.	Patients with multidirectional instability.
High-demand patients (Contact sports athletes and heavy labour workers).	Bilateral shoulder dislocation.
Age group from 18 to 45 years.	Patients with associated rotator cuff tear.
Glenoid bone loss < 15% of glenoid width.	Patients with associated fractures of the greater tuberosity or the proximal humerus.
Hill-Sachs lesion < 20% of humeral articular arc.	Patients with hyperlaxity (scoring > 5 according to Beighton score).
	Patients who previously underwent ipsilateral shoulder surgery for instability.

All patients were males with a mean age at surgery of 28.6 years (range, 18-41 years). There were 27 dominant (90%) and three non-dominant (10%) shoulders. The number of preoperative dislocation episodes ranged from two to eight with a mean of 4.7 dislocations. Twenty-one patients were heavy labour (70%), five were recreational athletes (13.2%) and four (16.6%) were professional athletes. The mechanism of the first dislocation was direct trauma in 12 patients (40%) and indirect trauma in 18 patients (60%).

Imaging

All patients had a preoperative plain X-ray, CT (computerised tomography) scan including “enface view” as well as an MRI (magnetic resonance imaging) scan. Glenoid bone loss was measured on the "en face" view of the 3D CT using the best-fit circle method of the affected shoulder. The size of the Hill-Sachs defect was calculated as a percentage of articular arc loss on axial MRI.

Surgical technique

Group A: Arthroscopic Bankart

Surgeries were done by a senior orthopaedic surgeon with all patients positioned in a 60-degree beach chair. A classic viewing posterior portal, anterosuperior and anteroinferior portals were established. An arthroscopic liberator was used to free up the scarred labrum from the glenoid anteriorly. Then, grasper was used to pull the labro-ligamentous complex to the articular margin while capsular tension and mobility are evaluated. The glenoid rim and scapular neck were then abraded to create a bleeding healing surface with an arthroscopic rasp, shaver or burr. A drill guide for the anchor was then introduced via the anteroinferior portal and was positioned over the glenoid's edge at 6 o’clock. Anchors were placed 1-2 mm onto the articular cartilage surface approximately 45° to the surface of the glenoid. A grasper was used to grip a limb of the anchor's suture and take it out via the anterosuperior portal. The capsulolabral tissue was then captured with a curved suture passer (lasso) loaded with a no. 1 polydioxanone (PDS) suture. The PDS was passed out of the lasso to the joint and then grasped out of the anterosuperior portal. A simple loop was then created with the PDS suture and loaded with the anchor suture and then cinched down by removing the suture passer and pulling the PDS limb shuttling the anchor suture via the tissue. A sliding knot followed by locking half hitches was applied. Subsequent anchors were placed in a similar fashion at 7.30 and 8.30 o’clock positions (left shoulder). Nonabsorbable sutures were then used to close the skin.

Group B: Open Latarjet

A deltopectoral approach was used with a 40-degree low beach-chair position. A total of 5-7 cm incision starting from the tip of the coracoid process directed downwards to the axillary fold. The cephalic vein and the deltoid were taken laterally exposing the conjoint tendon. With the arm in abduction and ER, the coracoacromial ligament (CAL) was best revealed and then incised. The arm was then positioned in internal rotation (IR) and adduction to expose the pectoralis minor muscle which was detached from the medial surface of the coracoid process. A coracoid osteotomy was performed at a line just anterior to the coracoclavicular ligament insertion at the coracoid base using a 90-degree oscillating saw (from medial to lateral) or a curved osteotome. The oscillating saw was then used to decorticate the inferior coracoid surface, exposing a broad ﬂat cancellous bone. Two drill holes were then made using a 2.7-mm drill bit in the central axis of the coracoid. The subscapularis muscle was split between the superior two-thirds and the inferior one-third. Capsule was then vertically incised and the anteroinferior labrum and periosteum were excised with electrocautery. The longitudinal axis of the coracoid graft was positioned supero-inferiorly along the glenoid neck flush with the articular surface. A 1.25-mm guide wire was then placed via the lower hole of the coracoid to maintain the position of the graft. The position of the coracoid graft is then checked. Then, the second 1.25 mm guide wire was placed via the superior hole. The inferior hole is then drilled with a 2.7 mm cannulated drillbit, a 35 mm long 3.5 cannulated screw is typically inserted in the inferior hole and intraarticular inspection is done to ensure that the screw is not penetrating glenoid articular cartilage. The superior hole was then drilled and a second 3.5 cannulated screw was inserted. The CAL stump was repaired to the capsule using sutures. Then, the subscapularis split was closed by absorbable sutures followed by layered closure of the rest of the wound.

Post-operative rehabilitation

All our patients had the same postoperative protocol. A shoulder immobilizer was applied for five weeks allowing passive and active assisted ROM exercises with no active ER or abduction above 90 degrees allowed. More aggressive strengthening in addition to active ROM was allowed after six weeks. Gradual return to sport/work was encouraged at 12 weeks.

Clinical outcomes

All patients were assessed pre- and post-operatively according to the Rowe scoring system as regards stability, motion and function of the shoulder. The rate, level and timing of return to sports/work were evaluated.

Statistical analysis

All statistical analysis was performed using Statistical Product and Service Solutions (SPSS) (IBM SPSS Statistics for Windows, Version 20.0, Armonk, NY). Continuous variables were displayed in range and mean ± SD and were statistically analysed using the student t-test and F-test (ANOVA) to compare the mean of the continuous variables. Pearson coefficient for correlation was used to correlate two continuous variables. Statistical significance was accepted when the p-value was less than 0.05.

## Results

Thirty patients met the inclusion criteria. The mean duration of follow-up in our study was 13.27 months±2.70. The overall mean Rowe score increased significantly from 33.5±14 points preoperatively to 79.6±18 points post-operatively. In the Bankart group, the mean Rowe score increased from 29±14 preoperatively to 74±18.8 and the mean ER at 90° abduction improved from 90° pre-operatively to 91° post-operatively. While the Latarjet group, the mean Rowe score increased from 38±12 pre-operatively to 85.3±15.8 post-operatively and the mean ER at 90° abduction remained the same post-operatively. However, there was no statistically significant difference in the postoperative ROM and the Rowe score among Bankart and Latarjet groups (Tables 2-4).

**Table 2:** Mean Rowe score in Bankart versus Latarjet

Rowe score	Bankart repair	Latarjet
Preoperative	29±14	38±12
Final follow-up	74±18.8	85.3±15.8

**Table 3:** The mean ROM in the Bankart group ROM: range of motion

Range of motion	Preoperative	Final follow-up
Forward Flexion	169°	170.7°
External Rotation at side	61.4°	61.9°
External Rotation at 90° abduction	90°	91°
Internal Rotation at 90° abduction	74.4°	74.7°

**Table 4:** The mean ROM in the Latarjet group ROM: range of motion

Range of motion	Preoperative	Final follow-up
Forward Flexion	172°	173.2°
External Rotation at side	62.8°	62.5°
External Rotation at 90° abduction	92.4°	92.4°
Internal Rotation at 90° abduction	75.2°	75.2°

The mean time to return to work/sport was 6.12 months. Eighteen patients (60%) managed to return to their pre-injury level and 10 patients (30%) patients returned to their sport/work but with limitations. Two patients (6.7%) changed their sport/work. There was a statistically significant difference between the mean time to return to work/sport for patients who underwent arthroscopic Bankart repair operation (seven months) compared to the Latarjet group (5.2 months). However, there was no difference between the two groups regarding the rate of return to work/sport.

Also, there was a statistically significant difference between the mean surgical time for arthroscopic Bankart repair (43.33±5.27 min) and the Latarjet procedure (72.33±10.38 min). Lastly, none of the patients had recurrent dislocation within the follow-up period.

## Discussion

In our study, we compared the results of Latarjet operation and arthroscopic Bankart repair in 30 high-demand patients including heavy labour and contact athletes with traumatic anterior shoulder instability. We excluded all the patients with glenoid bone loss > 15%, Hill-Sachs lesion > 20% of humeral articular arc as well as patients who had previous surgery for shoulder instability. The mean duration of follow-up was 13.27 months±2.70.

There is controversy in literature in terms of returning to sports post-Latarjet vs arthroscopic Bankart. Multiple studies showed rates of 66-100% for the return to pre-injury level post-Bankart operation [15-17]. Similarly, several studies showed that athletes who had the Latarjet operation returned to the same competition level at similar rates of 65-96% [18-20].

Jeon et al. [21] found no significant difference in returning to the same level of sports among Latarjet (96.8%) and Bankart (94.1%) concluding that the level of postoperative return to sports is not affected by the surgical method. Also, Bessière et al. [9] reported that 63% of patients who underwent the Bankart repair and 72% who underwent the Latarjet procedure returned to their post-injury sports at the same levels, although the difference was not statistically significant. Furthermore, a meta-analysis and systematic review is done by Ialenti et al. [22] reported no significant difference in the pre-injury level of return back to sports for athletes managed with Latarjet procedure or arthroscopic Bankart.

In our study, there was no statistical significance between the rate of return to sports/work in both groups. In the Latarjet group, 10 patients (66.7%) returned to the pre-injury level compared to eight patients (53.3%) in the Bankart group. The main finding of our study was the time to return to work/sports which was significantly lower in the Latarjet group (5.2 months) compared to the Bankart group (seven months).

Dekker et al. [23] showed no statistically significant difference in return to the competitive rates as well as the return time for athletes who had Bankart repair in comparison to the Latarjet procedure (4.6 vs 4.2 months). Ialenti et al. [22] showed in their systematic review that patients who underwent Bankart repair on average took approximately one month longer to return to sports compared with those who underwent the Latarjet procedure (6.1 vs 5.3 months).

Abdul-Rassoul et al. [24] assessed the time required for players to go back to the sport after various surgical options for anterior instability of the shoulder. Return to sports occurred at a mean of 5.9 months post arthroscopic Bankart, five months post open Latarjet and 5.8 months post arthroscopic Latarjet which is comparable to our aforementioned results.

All patients in our study were assessed according to the Rowe scoring system as regards stability, ROM and function of the shoulder. There was a statistically significant increase in the Rowe score postoperatively with a mean score of 79.6±18 points post-operatively compared to 33.5±14 points preoperatively. However, there was no statistically significant difference neither in Rowe's score postoperatively nor postoperative ROM between Bankart and Laterjet groups.

Ialenti et al. [22] found in their systematic review that the Rowe score was the most commonly reported patient outcome measure among the papers evaluated with the mean score for open Bankart was 86 postoperatively compared to 79.5 for arthroscopic Bankart and 82.0 for Latarjet groups concluding that there was no statistical difference among the three groups. Jeon et al. [21] found no significant difference in the clinical outcomes in arthroscopic Bankart and open Latarjet groups for patients managed for anterior instability with a borderline (15%-20%) glenoid defect.

Various studies stated that the Latarjet procedure could result in less limited ER compared to arthroscopic Bankart repair. Hovelius et al. [25] reported that patients for whom the Latarjet surgery was done had an 11-degree loss in ER, while those with Bankart surgery had a 19-degree loss. In a systematic review done by An et al. [10] comparing Bankart and Latarjet, the mean ER losses were 20.9 degrees and 11.5 degrees post-Bankart and Latarjet, respectively. Jeon et al. [21] showed in their study that loss of ER at the side was significantly increased post-Bankart repair (13.3 degrees) compared to Latarjet (7.3 degrees) justifying that excessive tension on the capsule was avoided in Latarjet by suturing the CAL to the mid-portion of the capsule, while Bankart repair puts tension on the capsule by pulling it as well as the labrum to their native position at the glenoid margin.

Recent studies have cited more recurrence of instability with Bankart particularly in young contact athletes encountering critical or subcritical glenoid loss, which has resulted in broadening the indications for such bone augmentation techniques, as Latarjet [3,26]. Jeon et al. [21] reported a recurrence rate of 22.9% (27 out of 118 shoulders) in the Bankart group compared to 6.5% (two out of 31 shoulders) in the Latarjet group within the final follow-up (mean of 28.9 months).

On the other hand, Ialenti et al. [22] found that recurrent dislocation was significantly less likely after Latarjet stabilization (3.5%) as compared with arthroscopic Bankart repair (6.6%) and open stabilization (6.7%). In our study, there were no recurrent dislocations which may be related to the short follow-up period and the small number of patients included in the study.

There are some limitations in our study. First, as mentioned above, the follow-up was short (13.27 months±2.70) and recurrence following surgery may happen between one and two years follow up, thus long-term follow-up is needed. Second, the number of subjects was small, but this was due to the precise inclusion criteria as we were targeting high-demand patients with a certain amount of bone loss. Last, given that our patients participated in different levels of work/sports activity, we endeavoured to confirm the return to pre-injury level as well as postoperative subjective satisfaction among our patients.

## Conclusions

Evidence-based data support the efficacy of both surgeries; arthroscopic Bankart and Latarjet, and the surgeons’ choice plays a major role in opting for one option over the other. Open Latarjet is considered a more invasive and non-anatomical procedure, however, it is less costly with a shorter time to return to sports/work compared to the Bankart procedure, which is very crucial for high-demand patients, especially the competitive athletes targeting an early return to sports at the same pre-injury level with minimal incidence of recurrence, making the surgeon’s choice very challenging.
